# Pretreatment with a ketogenic diet inhibits mitochondrial damage in rat models of spinal cord injury via the PGC-1α/Sirt1/Nrf1 pathway

**DOI:** 10.22038/ijbms.2025.82683.18565

**Published:** 2025

**Authors:** Xiaomeng Wang, Weibin Lan, Yang Liao, Haichuan Lu

**Affiliations:** 1 Department of Spinal Surgery, Affiliated Longyan First Hospital of Fujian Medical University, Longyan, 364000, Fujian, China; 2 Department of Radiology, Affiliated Longyan First Hospital of Fujian Medical University, Longyan, 364000, Fujian, China

**Keywords:** Apoptosis, Ketogenic diet, Mitochondria, Sirtuin 1, Spinal cord injury

## Abstract

**Objective(s):**

Spinal cord injury (SCI) often results in poor recovery prospects and a high disability rate. Although the ketogenic diet (KD) was suggested as a neuroprotective agent after SCI, its underlying mechanism remains unclear.

**Materials and Methods:**

Rats were divided into three groups: sham-operated controls, SCI with standard diet (SD), and SCI with KD. Following a 2-week dietary pretreatment, the SD and KD groups underwent a cervical level 5 hemi-contusion injury, and their neuromotor functions were monitored for 28 days. The expression levels of PGC-1α, Sirt1, Nrf1, and TOMM20 in the spinal cord tissues were measured using qPCR and immunofluorescence staining.

**Results:**

Compared with the SD group, KD pretreatment significantly improved neuromotor recovery and reduced neuronal apoptosis. The expression levels of PGC-1, Sirt1, and Nrf1 in the spinal cord tissues of rats in the KD group were significantly up-regulated. Additionally, KD was found to alleviate neuronal mitochondrial dysfunction by regulating TOMM20 expression.

**Conclusion:**

KD pretreatment enhances SCI recovery by modulating the PGC-1α/Sirt1/Nrf1 pathway, improving mitochondrial function, and reducing neuronal death. The study provides new insights into the mechanisms of KD.

## Introduction

Spinal cord injury (SCI) is predominantly caused by violent factors and has profound impacts on patients’ physical and mental health as well as professional development (1). Primary mechanical trauma leads to neuron death and disrupts the blood-spinal cord barrier. Secondary injury mechanisms such as inflammation, ischemia, oxidative stress, and mitochondrial damage propagate damage to initially spared tissue, resulting in motor and sensory dysfunctions (2-4). SCI is a debilitating condition with limited treatment options. Current clinical treatment protocols are based on early surgical decompression and pharmacotherapy. Techniques such as tendon transfer, tenodesis, and arthrodesis (often used in combination) are well**-**established strategies for enhancing motor function (5). Pharmacologic therapy for acute SCI includes corticosteroids, erythropoietin, Granulocyte Colony-Stimulating Factor (G-CSF), Hepatocyte Growth Factor (HGF), opioid antagonists, Cethrin, minocycline, Riluzole, and GM-1 ganglioside (Sygen) (6). However, the efficacy of these interventions remains suboptimal. Pharmacological agents often address symptom management but fail to reverse neurological deficits. There is an urgent need for novel therapies targeting secondary injury mechanisms, such as mitochondrial dysfunction, oxidative stress, and apoptosis, to enhance neuroprotection and promote functional recovery.

The ketogenic diet (KD) is a high-fat, low-carbohydrate diet with an adequate yet variable amount of protein (7,8). KD induces a metabolic state characterized by fatty acid oxidation and hepatic ketogenesis, resulting in an increase in the concentration of β-hydroxybutyrate (BHB) in the blood. Ketogenic metabolism has been employed to treat pediatric intractable epilepsy (9). A number of clinical and pre-clinical studies have proved the beneficial effects of KD in neurodegenerative diseases such as amyotrophic lateral sclerosis, cerebral ischemia, traumatic brain injury, Alzheimer’s disease, and Parkinson’s disease (10-13). Furthermore, the neuroprotective role of ketones has been shown through improved histological and behavioral outcomes in rats following acute SCI for both the KD and every-other-day-fasting regimes (7, 14, 15). While previous studies have demonstrated the neuroprotective effects of KD in SCI, the precise molecular mechanisms underlying these benefits, particularly regarding mitochondrial function and the associated signaling pathways, remain incompletely understood.

Accumulating evidence indicates that the ketone metabolite BHB has a variety of signaling functions. These include suppressing oxidative stress by inhibiting class I histone deacetylases, enhancing adenosine 5’-monophosphate (AMP)-activated protein kinase (AMPK) activity, and modulating autophagy (16-18). Notably, AMPK is the foremost regulator of mitochondrial bioenergetics. Some evidence also links mitochondrial function to the actions of BHB. In mitochondria, BHB can be converted to acetoacetate, generating NADH and, by further cleavage, acetyl-CoA (19). Administration of BHB can stabilize complex II activity, raise succinate concentrations, and decrease reactive oxygen generation (20,21). Moreover, a study by Lehto and colleagues demonstrated that the ketone metabolite BHB improved mitochondrial function after transient ischemia (22). In the current study, we specifically investigated the involvement of the PGC-1α/Sirt1/Nrf1 pathway in KD-mediated neuroprotection after SCI. Our findings demonstrate that KD is a potential preventive strategy targeting mitochondrial dysfunction, a key contributor to secondary injury. This advances the field by identifying specific targets (e.g., Sirt1, PGC-1α) for future interventions.

## Materials and Methods

### Animals and grouping

Sprague-Dawley rats are commonly used as an animal model for preclinical SCI studies. Male Sprague–Dawley rats (8 weeks old, 290–320 g) were housed five per cage under controlled conditions (temperature: 22 ± 1 °C; humidity: 55 ± 5%; 12 hr light/dark cycle) with *ad libitum* access to food and water*. *Animals were acclimatized for seven days prior to experiments. A total of 54 rats were randomly divided into three groups: a sham-operated (Sham) group; a standard diet (SD) group fed a regular carbohydrate-based rodent diet provided by the Experimental Animal Center of Affiliated Longyan first Hospital of Fujian Medical University; and a KD group that was given a diet with a ratio of 3.1:1 for fat to carbohydrate and protein, supplied by Trophic Animal Feed High-Tech Co., Ltd, China. The essential nutrients of SD and KD are presented in Table 1. After two weeks, rats in both the SD and KD groups underwent SCI surgery. All experiments were approved by the Clinical Ethics Committee of Longyan First Hospital (No. LYEC-2022-204) and complied with the National Research Council’s Guide for the Care and Use of Laboratory Animals. The research adhered to the ARRIVE guidelines for reporting *in vivo* experiments.

### Surgical procedures

A previously outlined C5 hemi-contusion model was utilized with certain alterations (23,24). Briefly, the rats were anesthetized with 2% isoflurane in oxygen. Unilateral laminectomy at C5 was carried out to uncover the spinal cord. A specially designed clamping system was used to hold the spinal column firmly in place between the C4 and C6 vertebrae. Subsequently, the rat was placed under a servo-electromagnetic material testing apparatus (ElectroPuls E1000; Instron, MA, USA). The cylinder impactor, having a flat head and a diameter of 1.5 mm, along with a rounded edge, was directed towards the C5 spinal cord at a location 1.4 mm to the left of the midline, as identified by the posterior spinal artery. It was triggered to deliver a set displacement of 1.6 mm at a velocity of 600 mm/s. Following the injury, the animals were placed in an incubator with a controlled temperature until they were fully awake. 

### Measurements of body weight and blood BHB

The body weight and blood BHB levels were measured before surgery, as well as at regular intervals throughout the experimental timeline. The glucose and BHB concentration (mM) of tail vein blood were assessed using an electrochemical BHB meter (Abbott Diabetes Care Ltd., Oxon, UK).

### Basso mouse scale (BMS) analysis

The evaluation of hindlimb motor function recovery was conducted using the Basso Mouse Scale (BMS), a standardized scoring metric ranging from 0 to 9. This comprehensive evaluation encompassed diverse criteria, such as lower limb joint mobility, coordination abilities, trunk stability, paw positioning, and tail posture. Preoperative motor functions were benchmarked, and evaluations were performed at several time points (1, 3, 7, 14, 21, and 28 days) subsequent to SCI.

### Footprint analysis

Twenty eight days after SCI, the gait and motor coordination of rats were evaluated through footprint analysis. The forelimbs and hindlimbs of the animals were stained with blue dye and red dye, respectively. When the mice walked in a straight line at a constant pace, the resultant footprints were recorded using a digital camera, and a specific set of representative images was identified for detailed examination.

### Nissl staining

On the 28th day post-SCI, spinal cord tissues from distinct experimental groups were subjected to Nissl staining. The spinal cord segments were removed and fixed in 4% paraformaldehyde. After 48 hr, the spinal cord sections from the caudal 5 mm areas, as well as the lesion location, were cut into 5-micron-thick slices transversely. These slices were then immersed overnight in a 1:1 ratio mixture of ethanol and chloroform. The slices were rinsed three times with PBS, immersed in a cresyl violet solution at 37 °C for 40 sec, and then washed once with distilled water. Before being mounted with neutral glue and observed under a light microscope, the slices were dehydrated using 95% ethanol, absolute ethanol, and finally xylene. To maintain objectivity, researchers who were unaware of the experimental groups examined the staining patterns in the damaged areas.

### TUNEL assay

Tissue sections were dried at ambient temperature for 30 min, then rinsed with PBS for 15 min, and permeabilized with 0.3% Triton X-100 for 10 min. Following a 2-hour blocking with sheep serum (Sigma), sections were incubated with NeuN (1:200, Proteintech). By employing the TUNEL detection kit (C1090, Beyotime, China), a TUNEL working detection solution was prepared, and subsequently, NeuN staining was performed using Alexa Fluor 568 (1:250; Thermo-Fisher Scientific, MA, USA). The remaining sections underwent immunofluorescent staining. After all sections were stained with DAPI (Invitrogen) for 15 min, the proportion of TUNEL-positive neurons was quantified using ImageJ.

### Immunofluorescence staining

Sections were processed with 0.3% TritonX-100 for 20 min, and subsequently had a 1-hour blocking step with 5% BSA at room temperature. The sections were incubated overnight at 4 °C with the corresponding primary antibodies. A secondary antibody (1:200) was added and incubated for one hour in the dark. Following three washes with PBS, the nuclei were stained with DAPI. The antibodies used in this experiment are: TOM20 (11802-1-AP, Proteintech, China), anti-MAP2 (8707T, Cell Signaling Technology, USA), and MitoTracker Red CMXRos (M7512, Invitrogen, USA). The fluorescence intensity was quantified using ImageJ.

### Real-time PCR analysis

Total RNA was extracted from neurons employing the TRIzol reagent (Invitrogen, USA) in accordance with the manufacturer’s protocol. The transformation of mRNAs to cDNAs was accomplished by using a reverse transcription kit (Takara, Japan). A quantitative real-time polymerase chain reaction (qRT-PCR) was then performed, making use of the SYBR Green reagent (Takara) and the primers listed below, with the cDNA serving as the template: β-actin, Forward: 5’-ACCCTAAGGCCAACCGTGA-3’,Reverse:5’-TGGCGTGAGGGAGAGCATA-3’; PGC-1α, Forward: 5’-GGCTCCTGCAAATGCAAACAATGC-3’, Reverse: 5’- CTGCACTTGTCCGAAGCCTCTTT-3’;SIRT1,Forward:5’-GCTGACGACTTCGACGACG-3’,Reverse:5’-TCGGTCAACAGGAGGTTGTCT-3’;Nrf1,Forward:5’-GGGCAAAGCAGACCCTCAAACT-3’,Reverse:5’-GTTAGGAAGATGGCGTGGGAGT-3’. The relative mRNA expression was determined by using the 2-∆∆Ct method.

### Western blotting

At a specific time point, the animals were perfused with normal saline, and approximately 2 cm segments were removed from the injured spinal cord. Protein was extracted from the segments by homogenizing them in RIPA lysis buffer, and its concentration was determined using the BCA assay. The proteins were separated by electrophoresis and transferred onto a membrane. Following blocking, the membrane was incubated overnight at 4 °C with primary antibodies. The used antibodies were: anti-PGC-1α (66369-1-Ig, Proteintech, China), anti-Sirt1(13161-1-AP, Proteintech, China), anti-Nrf1(ab175932, Abcam, UK), anti-Beta Actin (66009-1-Ig, Proteintech, China). Following a 1-hour incubation with the secondary antibody at room temperature, the protein bands were detected using ECL hypersensitive luminous solution, and the fluorescence intensity was analyzed.

### Transmission electron microscopy analysis

The sample was initially prefixed with 3% glutaraldehyde, followed by refixation with 1% osmium tetroxide, and then dehydrated with acetone. Once embedded in Ep812, the sample was cut into slices, dyed with uranium acetate and lead citrate. It was finally observed under a JEM-1400FLASH transmission electron microscope.

### Statistical analysis

Analyses were performed using SPSS 20.0 software (IBM, NY, USA). The data were shown as mean ± standard deviation. The one-way analysis of variance (ANOVA), along with the least significant difference *post hoc* test, was used for comparisons among multiple groups. For multiple group comparisons at various time points, multivariate analysis of variance (MANOVA) with repeated measures was utilized. Statistical significance was set at *P*<0.05.

## Results

### KD increased blood β-hydroxybutyrate Levels Compared with standard diet

**Figure 1:**
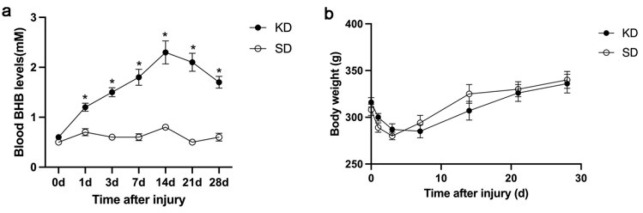
Body weight and blood BHB levels in sprague-dawley rats were assessed at various time points

The blood BHB levels of the KD group were significantly higher than those of the SD group (*P*<0.05, Figure 1a), peaking at 2.3 mM after 2 weeks of KD feeding and maintaining a consistently high level throughout the study. Conversely, the blood BHB levels in the SD group fluctuated between 0.3 and 0.6 mM. There was no significant difference in animal body weight between the KD group and SD group at any of the inspected time points (Figure 1b).

### KD promoted the recovery of motor function in mice with SCI

**Figure 2:**
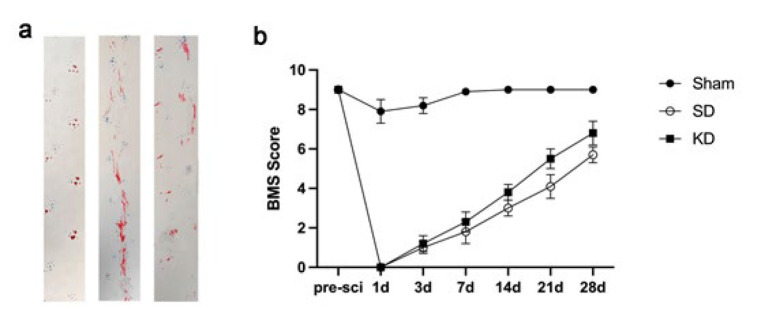
Effects of KD on the restoration of motor function in sprague-dawley rats with SCI

We evaluated the impact of KD on motor function rehabilitation using behavioral analysis. Gait analysis revealed that the rats’ hindlimbs stepped on the ground with a clear and steady stride. Sham group rats exhibited the longest stride length (Figure 2a). By contrast, the rats in the SCI group demonstrated a trailing gait accompanied by a shorter stride. Notably, the stride length of the hindlimbs in the KD group was significantly longer than that in the SD group, suggesting improved motor function. Consistent with this, BMS scores also indicated the positive effect of KD on motor recovery (Figure 2b).

**Figure 3:**
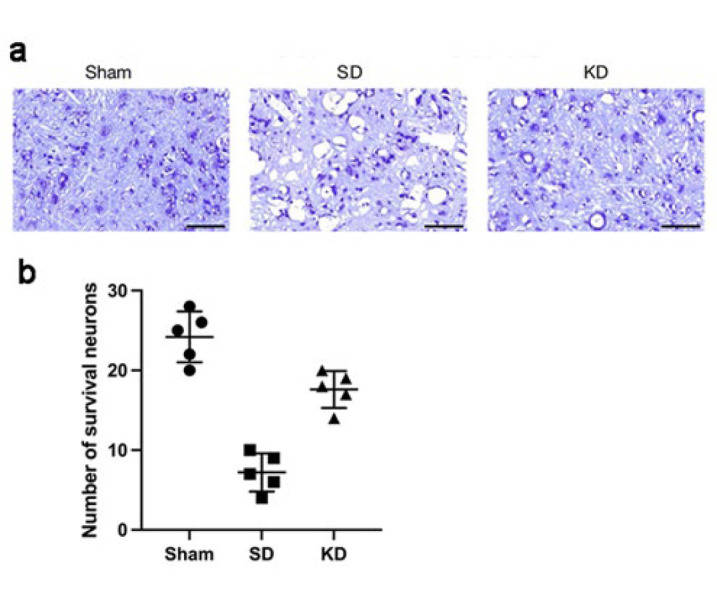
Effects of KD on the neurons in sprague-dawley rats with SCI

Nissl staining served as a morphological indicator for assessing neuronal activity. We performed this experiment to determine the count of neurons near the injury site at 28 days post-SCI. Our observations revealed that the KD group exhibited a significantly higher motor neuron count compared to the SD group, albeit lower than that of the sham-operated group, suggesting enhanced neuronal functional activity (Figure 3a and b).

### KD alleviated mitochondrial-related neuronal apoptosis after SCI

**Figure 4:**
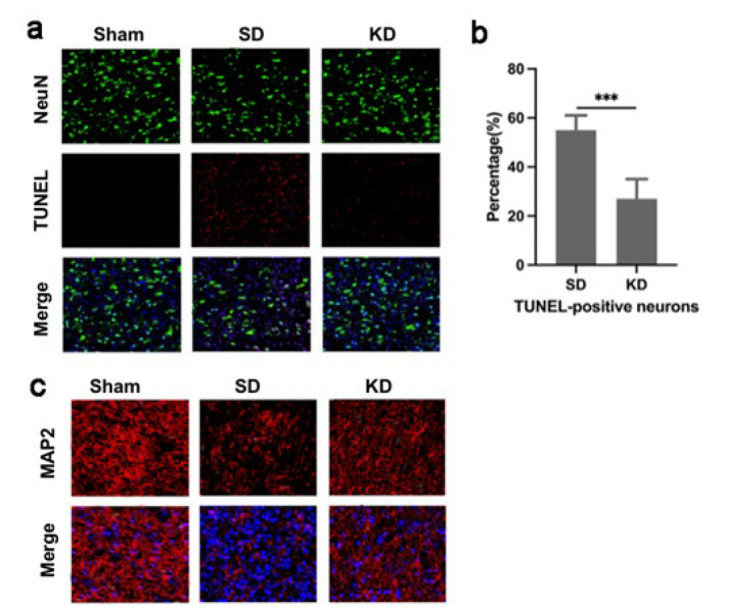
Effects ofKDtreatment on the apoptosis of neurons in s p r a g u e - d a

Next, TUNEL staining was performed to detect indicators of neuronal apoptosis in spinal cord slices. Following SCI, a significant number of apoptotic neurons were observed, and KD notably decreased the proportion of TUNEL-positive neurons (Figures 4a and b). MAP2 marks neuronal cell bodies and axons. We found that KD was able to raise the expression of MAP2 at 28 days post-injury (Figure 4c), aligning with the pattern of KD-mediated inhibition of neuronal apoptosis.

*KD inhibited mitochondrial damage in mice with SCI.*

**Figure 5:**
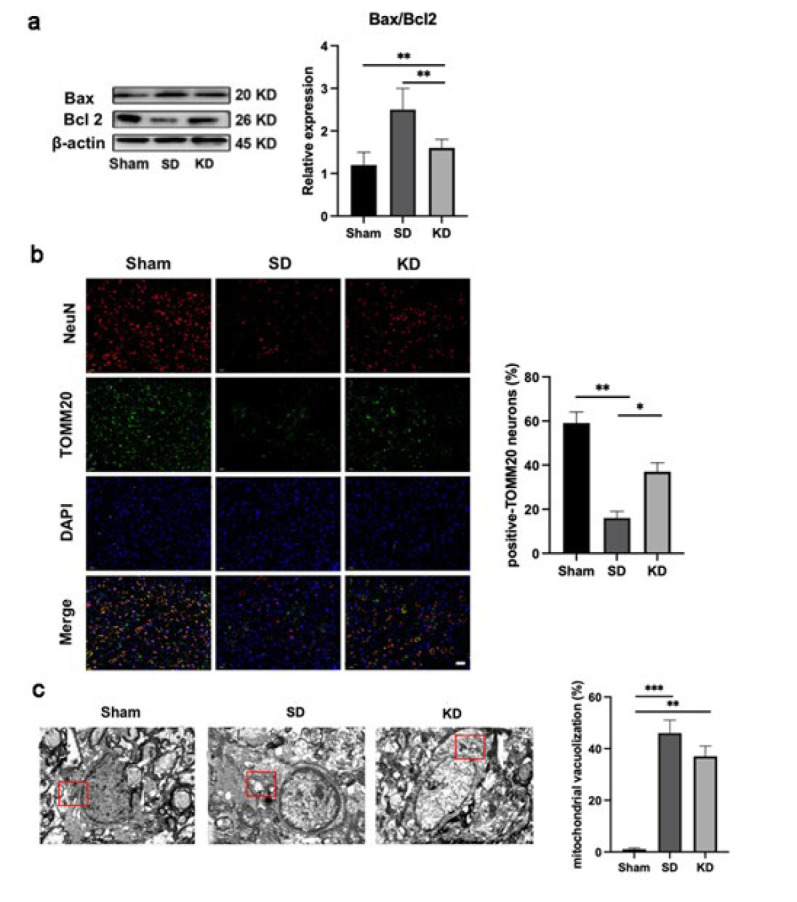
KD treatment attenuated mitochondrial-associated neuronal apoptosis in sprague-dawley rats

We investigated the potential link between neuronal apoptosis and mitochondrial dysfunction. Bax, a protein that promotes mitochondrial membrane permeabilization, thereby triggering apoptosis, was up-regulated after SCI. Meanwhile, Bcl-2, which prevents the increase in mitochondrial membrane permeability, was downregulated after SCI. This trend was reversed following KD intervention (Figure 5a).

TOMM20 served as a marker for labeling mitochondria and analyzing continuous mitochondrial structures (with a size greater than 2 μm), indicative of functional mitochondria (25). The immunofluorescence staining revealed that KD treatment enhanced the expression of TOMM20 (Figure 5b).

Transmission electron microscopy provided insight into the ultrastructural changes in mitochondria of the spinal cord. Notably, the mitochondria were impaired after SCI, characterized by increased vacuolation. By contrast, rats with SCI and who received KD treatment exhibited a decreased number of vacuoles, and the mitochondrial cristae were restored as observed (Figure 5C).

### KD activated Sirt1 and enhanced the PGC-1ɑ signaling after SCI

**Figure 6:**
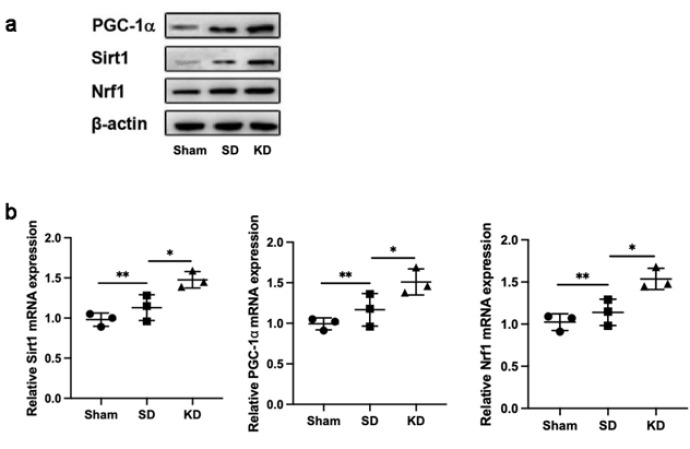
KD activated the PGC-1α/Sirt1/Nrf1 pathway following SCI in sprague-dawley rats

Western blot (WB) assessment revealed that, in comparison to the SD group, the amounts of PGC-1α, Sirt1, and Nrf1 expression were markedly elevated in the KD group (Figure 6a). Moreover, in harmony with the WB observations, similar expression patterns of PGC-1α, Sirt1, and Nrf1 within neurons across the three groups were confirmed by qRT-PCR (Figure 6b).

## Discussion

In this study, we demonstrated that pretreatment with KD significantly inhibits neuronal apoptosis and alleviates mitochondrial dysfunction following SCI in rats. Our findings showed that these neuroprotective effects were mediated, at least in part, by activation of the PGC-1α/Sirt1/Nrf1 signaling pathway, which plays a pivotal role in restoring mitochondrial bioenergetics and cellular homeostasis post-injury.

In the context of SCI, two distinct pathological stages emerge: the primary injury and secondary injury. Primary injury is typically irreversible, whereas secondary injury possesses the potential for reversibility. The latter is characterized by cascades of inflammation, oxidative stress, and mitochondrial dysfunction, ultimately leading to neuronal apoptosis—a major barrier to spinal cord repair. Mitochondria are central to this process, as they fuel critical repair mechanisms including calcium signaling, cytoskeletal reorganization, and anti-oxidant responses (26). Our findings align with previous reports showing that ketone bodies can preserve mitochondrial function in various neurological conditions, including traumatic brain injury and neurodegenerative diseases (10,11,27). 

Mechanistically, KD elevated the expression of PGC-1α, Sirt1, and Nrf1 compared to standard diet-fed rats, suggesting activation of a coordinated mitochondrial biogenesis program. In our study, both the SD and KD groups exhibited elevated expression of PGC-1α, Sirt1, and Nrf1 compared to sham controls, indicating that the PGC-1α/Nrf1/Sirt1 pathway was activated following injury. This is likely to be a compensatory response to injury. KD further amplified this effect, correlating with improved mitochondrial function and reduced apoptosis. This suggests that KD not only augments endogenous repair mechanisms but also provides additional metabolic support to counteract secondary injury.

Sirt1 acts as a downstream signaling effector molecule of AMPK, boosting PGC-1α activity through deacetylation. PGC-1α is a critical regulator of mitochondrial bioenergetics (28,29). It has been reported that the activation of the Sirt1 pathway was crucial for effectively inhibiting apoptosis and autophagy in nerve cells following SCI, consequently reducing disability resulting from the injury (30). The overexpression of PGC-1α drives the transition from glycolytic to oxidative skeletal muscle and up-regulates respiratory chain complexes (complexes II, IV, and V at the mRNA level; complexes I and IV at the protein level) (31,32). PGC-1α knockout reversed this transformation (33,34). Certain therapeutics, such as rosmarinic acid, have been demonstrated to enhance mitochondrial bioenergetics through the Sirt1 pathway activation, but their clinical translation remains challenging due to pharmacokinetic limitations (35). In contrast, KD offers a clinically feasible, multi-targeted approach to enhance mitochondrial function. Moreover, KD provides a clinically viable strategy to augment endogenous repair mechanisms and improve functional recovery after SCI. 

KD requires a sustained period to induce systemic ketosis in rodents, ranging from 10 days to 4 weeks before the commencement of experiments (36). Our data showed stable elevation of blood β-hydroxybutyrate levels after 2 weeks of dietary intervention. Notably, the initial phase of ketosis is associated with transient spikes in adenosine levels, which may confer short-term neuroprotective effects through anti-inflammatory and metabolic modulation (37). However, the translational implications of these findings warrant careful consideration. Adult humans exhibit a greater capacity to utilize ketones for energy metabolism (fulfilling up to 60% of metabolic demand compared to ~25% in rodents), suggesting that KD’s therapeutic efficacy may be more pronounced in clinical settings than in rodent models (38-40). Preclinical studies further corroborate that KD regimens enhance motor recovery, gray matter sparing, and pain thresholds in rodent SCI models (41, 42). Supporting this premise, a pilot randomized clinical trial demonstrated that KD initiation within 72 hr post-SCI was well tolerated and associated with anti-inflammatory effects and improved neurological recovery (43). Nevertheless, clinical adoption of KD faces challenges, including dietary complexity and patient adherence, highlighting the need for alternative strategies such as ketone supplements to sustain therapeutic ketosis without stringent dietary restrictions.

While our study and others have demonstrated a role of ketone bodies in mitigating neuroinflammation, oxidative stress, and mitochondrial dysfunction, the precise molecular mechanisms, particularly regarding the temporal and spatial regulation of PGC-1α/Sirt1/Nrf1 signaling, require further investigation. Future studies should explore the therapeutic potential of post-injury KD administration, rather than preventive approaches, to better align with clinical scenarios. Additionally, longitudinal monitoring of ketosis states in relation to functional recovery outcomes could provide valuable insights into optimal treatment duration and metabolic requirements. The development of ketone mimetics may also offer a promising alternative to circumvent the practical challenges of long-term dietary interventions. Such advances will be crucial for elucidating the underlying mechanisms to support clinical application of KD and accelerate the design of targeted therapies for SCI.

**Table 1:** Basic nutrient content of SD and KD for sprague-dawley rats (per100g)

Component	SD	KD
Energy (kJ)	1338.0	2804.0
Protein (g)	14.5	18.2
Fat (g)	4.0	65.1
Carbohydrates (g)	55.5	2.7
Dietary fibers (g)	4.5	7.4
Calcium (mg)	720.0	500.0
Phosphorus (mg)	600.0	300.0
Vitamin D (μg)	2.5	2.5

Ratio of fat to carbohydrate and protein in KD is 3.1:1.

SD: Standard diet; KD: Ketogenic diet.

(a) The levels of blood BHB in the KD group were significantly higher than those in the SD group following SCI. (b) There was no significant difference in animal weight between the KD and SD groups at any of the time points under examination. Data represent the mean ± standard deviation (n=5). * *P*<0.05.

BHB: β-hydroxybutyrate; KD: Ketogenic diet; SD: Standard diet; SCI: Spinal cord injury

(a) Characteristic footprints of walking 28 days post-SCI and quantifying the outcomes of footprint analysis for individual rats. Blue: forelimbs; Red: hindlimbs. (b) BMS score was employed to evaluate motor functional recovery in different groups of rats from pre-injury to 28 days post-injury (n=10).

BHB: β-hydroxybutyrate; KD: Ketogenic diet; SD: Standard diet; SCI: Spinal cord injury; BMS: Basso mouse scale

(a) Nissl staining was conducted to exhibit the neurons of rats in each group. (b) Quantified results of the number of survival motor neurons in every group (n=5).

KD: Ketogenic diet; SCI: Spinal cord injury

wl e y ratswith SCI

(a) NeuN (red) represents neurons, TUNEL (green) indicates DNA fragments after apoptotic processes, and DAPI (blue) indicates nuclei. The scale bar is 100 μm. (b) Shown is the statistical analysis of TUNEL-positive neuronal cells (n=5). ***P<0.001. (C) Neurons were stained with MAP2, and nuclei were marked with DAPI (in blue). The scale bar is 50 µm.KD: Ketogenic diet; SCI: Spinal cord injury; DAPI: Diamidino phenylindole

(a) Western blot analysis was performed to quantify the levels of Bax/Bcl-2 in each group (n = 5). (b) Immunofluorescence analysis confirmed the presence of TOMM20-positive neurons in the spinal cord tissue of mice with SCIs. Immunofluorescence images show NeuN (red), TOMM20 (green), and DAPI (blue). The scale bar is 100 μm. Statistical analysis of the neurons positive for TOMM20 (n=5). (c) Mitochondrial damage in the spinal cord tissue of rats with SCI was detected using TEM at 7 days post-injury, and the statistical analysis of the mitochondrial vacuolation rate was performed (n=5). *P<0.01, **P<0.05, ***P<0.001.

KD: Ketogenic diet; Spinal cord injury

(a) Western blot was performed to analyze he expression levels of PGC-1α, Sirt1, and Nrf1 in each group (n=3). (b) qRT-PCR was employed to confirm the expression of target genes. *P<0.01, **P<0.05.

KD: Ketogenic diet; SCI: Spinal cord injury

## Conclusion

In summary, this research indicated the role of pretreatment with KD in modulating the PGC-1α/Sirt1/Nrf1 signaling pathway, which is critical for mitochondrial functions in SCI. This work elucidated a mechanistic pathway through which KD exerts its neuroprotective effects, thereby providing a foundation for more targeted and effective treatments for SCI.

## Declaration

We have not used any AI tools or technologies to prepare this manuscript.
